# Supplementary material for: Simultaneous learning of static and dynamic charges
Source: Phys Chem Chem Phys. 2026 Jun 18;28(26):16249–58. doi: 10.1039/d6cp00911e (PMC13276636; doi:10.1039/d6cp00911e)
Supplement: CP-028-D6CP00911E-s001 [file CP-028-D6CP00911E-s001.pdf]

Supplementary Information (SI) for Physical Chemistry Chemical Physics.
This journal is © the Owner Societies 2026

# Supplemental Information: Simultaneous Learning of Static and Dynamic Charges

Philipp Stärk,[1,2] Henrik Stooß,[3] Marcel F. Langer,[4] Egor Rumiantsev,[4]
Alexander Schlaich,[3,*] Michele Ceriotti,[4,†] and Philip Loche[4,5,6,7,‡]

[1] *Stuttgart Center for Simulation Science (SC SimTech), University of Stuttgart, 70569 Stuttgart, Germany*
[2] *Institute for Computational Physics, University of Stuttgart, 70569 Stuttgart, Germany*
[3] *Institute for Physics of Functional Materials, Hamburg University of Technology, 21073 Hamburg, Germany*
[4] *Laboratory of Computational Science and Modeling, IMX, École Polytechnique Fédérale de Lausanne,1015 Lausanne, Switzerland*
[5] *Department of Physics, Technical University of Munich, 85747 Garching, Germany*
[6] *Atomistic Modelling Center, Technical University of Munich, 85747 Garching, Germany*
[7] *Munich Center for Machine Learning (MCML), 80538 Munich, Germany*

## I. CALCULATING BECS FROM LEARNED CHARGES IN PERIODIC SYSTEMS: IN PRINCIPLE

In the main text, we indicated that care has to be taken in using infrared charges $q^i_{\mathrm{IR}}$ for the calculation of $Z^i_{\alpha\beta}$ in systems with periodic boundary conditions (pbc). The reason for this is that in pbc, particle positions are only defined up to integer multiples $n_a$ of the lattice vectors $\boldsymbol{c}_a$. In other words, the arbitrary choice of which unit cell to pick and where to draw its boundary can add or remove offsets from $\boldsymbol{r}_j$. This changes the value of $\boldsymbol{P}$, and, by the chain rule, also for its derivatives $Z^i_{\alpha\beta}$ when using the "naive definition" of $\boldsymbol{P} = \sum_j q_j \boldsymbol{r}_j$. Let us consider the case where the boundary is shifted such that only one position, $i$, crosses it:

$$\boldsymbol{r}_j \longrightarrow \boldsymbol{r}_j + \sum_{a=1}^{3} n_a \boldsymbol{c}_a \tag{S1}$$

This changes the polarization

$$\sum_j q_j \boldsymbol{r}_j \longrightarrow \sum_j q_j \boldsymbol{r}_j + q_i \sum_{a=1}^{3} n_a \boldsymbol{c}_a \tag{S2}$$

and consequently the derivatives

$$\frac{\partial}{\partial \boldsymbol{r}_k} \sum_j q_j \boldsymbol{r}_j \longrightarrow \frac{\partial}{\partial \boldsymbol{r}_k} \sum_j q_j \boldsymbol{r}_j + \left( \frac{\partial}{\partial \boldsymbol{r}_k} q_i \right) \sum_{a=1}^{3} n_a \boldsymbol{c}_a \tag{S3}$$

in an arbitrary way that the model cannot learn and leads to noisy predictions observed by Zhong et al. [1].

We propose to fix this ambiguity by transitioning to relative, boundary-invariant, positions for the $\boldsymbol{r}_j$ used in constructing $\boldsymbol{P}$. To motivate this, we note that since $\sum_j q_j = 0$ due to charge neutrality, an offset can be added to $\boldsymbol{r}_j$ without impacting $\boldsymbol{P}$. For the choice of offset, we exploit the construction of the model that predicts $q_j$ from atomic positions: To ensure both translational and boundary invariance, the charge at position $j$ depends only on relative positions $(\boldsymbol{r}_j - \boldsymbol{r}_i)_{\mathrm{PBC}}$ that point, crucially, *not* neccessarily to the position $i$ located in the unit cell. Rather, $(\boldsymbol{r}_j - \boldsymbol{r}_i)_{\mathrm{PBC}}$ always indicates the *closest* replica. In small cells, there may also be multiple relative positions pointing to different replicas of the same original position. This definition of the BEC is then

$$\frac{\partial P_\alpha}{\partial r_{i,\beta}} = q^i_{\mathrm{IR}} \delta_{\alpha,\beta} + \sum_j \frac{\partial q^j_{\mathrm{IR}}}{\partial r_{i,\beta}} \left( r_{j,\alpha} - r_{i,\alpha} \right)_{\mathrm{PBC}}, \tag{S4}$$

*Electronic address: alexander.schlaich@tuhh.de; Authors contributed equally to this work.
†Electronic address: michele.ceriotti@epfl.ch; Authors contributed equally to this work.
‡Electronic address: philip.loche@epfl.ch; Authors contributed equally to this work.

which is boundary invariant. The same expression can be obtained as the $k \to 0$, i.e., large box, limit of the expression by Zhong et al. [1]:

$$q_i \delta_{\alpha,\beta} + \mathrm{Re}\left\{ \sum_j \frac{\partial q_j}{\partial r_{i,\beta}} \frac{\exp(ik(r_{j,\alpha} - r_{i,\alpha}))}{ik} \right\}. \tag{S5}$$

## II. CALCULATING BECS FROM LEARNED CHARGES IN PERIODIC SYSTEMS: IN PRACTICE

While eq. (S4) is simple in principle, its practical implementation requires additional discussion. This is because as written, it requires explicit access to the partial derivatives $\frac{\partial q^j_{\mathrm{IR}}}{\partial r_{i,\beta}}$: For each pair of $i$ and $j$, a vector $(\boldsymbol{r}_j - \boldsymbol{r}_i)_{\mathrm{PBC}}$ must be assigned. This amounts to evaluating the Jacobian of a multi-valued function (the one that predicts $q_j$) with automatic differentiation, which requires $N$ backward-mode evaluations or $3N$ forward-mode evaluations, neither of which is computationally feasible.

A similar problem occurs in the evaluation of the heat flux for machine-learning interatomic potentials. A solution was presented by Langer et al. [2, 3], based on two ideas:

First, we do not work in pbc, but in an extended system that explicitly constructs all relevant replica positions. This construction allows us to split the derivative $\frac{\partial q_j}{\partial r_{i,\beta}}$ into a sum over all the explicit replicas of $i$ that contribute to $q_j$. Letting a prime index indicate explicitly extended, "ghost" positions:

$$\frac{\partial q_j}{\partial r_{i,\beta}} = \sum_{i'} \frac{\partial q_j}{\partial r_{i',\beta}}. \tag{S6}$$

Since the $\boldsymbol{r}_{i'}$ already respect pbc,

$$(\boldsymbol{r}_j - \boldsymbol{r}_{i'})_{\mathrm{PBC}} = \boldsymbol{r}_j - \boldsymbol{r}_{i'}; \tag{S7}$$

we no longer require a peculiar measure of the distance between $i$ and $j$. For local (or semi-local) models, constructing the extended system is feasible as only a small shell of additional positions around the unit cell are required.

Second, we construct an auxiliary quantity $B_\alpha := \sum_j \hat{r}_{j,\alpha} q_j$ where the positions $\hat{r}_{j,\alpha}$ are *excluded* from automatic differentiation. Thus, the gradient of $\boldsymbol{B}$ with respect to positions yields

$$\frac{\partial B_\alpha}{\partial r_{i',\beta}} = \sum_j r_{j,\alpha} \frac{\partial q_j}{\partial r_{i',\beta}}. \tag{S8}$$

Putting it all together, we can write $Z^i_{\alpha,\beta}$ as:

$$Z^i_{\alpha,\beta} - q_i \delta_{\alpha,\beta} = \sum_j \frac{\partial q_j}{\partial r_{i,\beta}} (r_{j,\alpha} - r_{i,\alpha})_{\mathrm{PBC}} \tag{S9}$$

$$= \sum_j \sum_{i'} \frac{\partial q_j}{\partial r_{i',\beta}} (r_{j,\alpha} - r_{i',\alpha}) \tag{S10}$$

$$= \sum_{i'} \underbrace{\frac{\partial B_\alpha}{\partial r_{i',\beta}}}_{3\text{ grad}} - \sum_{i'} r_{i',\alpha} \underbrace{\frac{\partial}{\partial r_{i',\beta}} \sum_j q_j}_{1\text{ grad}}, \tag{S11}$$

which requires only the evaluation of the gradient of $B_\alpha$ and of $\sum_j q_j$, a total of four backward-mode evaluations with automatic differentiation.

Given that all information needed to explicitly include periodic replicas are inherently given by the neighborlist, implementing eq. (S11) is relatively straightforward. Thus, we expect an additional factor in the computational cost of the coupled method relative to uncoupled approaches of $\approx 4$.

This expected factor is visible in the benchmark shown in fig. S1, where both the coupled and uncoupled architecture approach the expected asymptotical scaling due to the Ewald sum of $\mathcal{O}(N^{3/2})$ with a difference between both architectures that approaches a factor of 3-4 for larger systems.

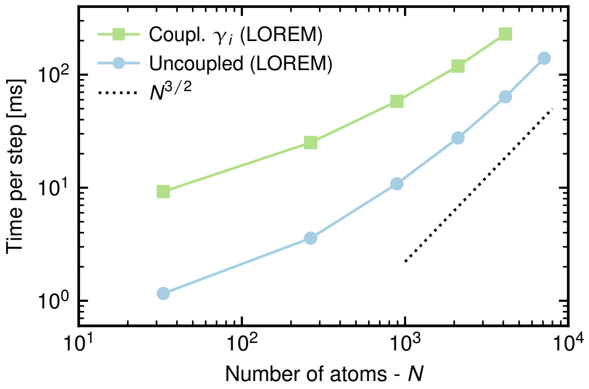


FIG. S1: Benchmark for computational cost of the LOREM models to run a single point evaluation of the energy, force, and BEC for a water structure with varying number of atoms. Each point is an average of 10 single point calculations.

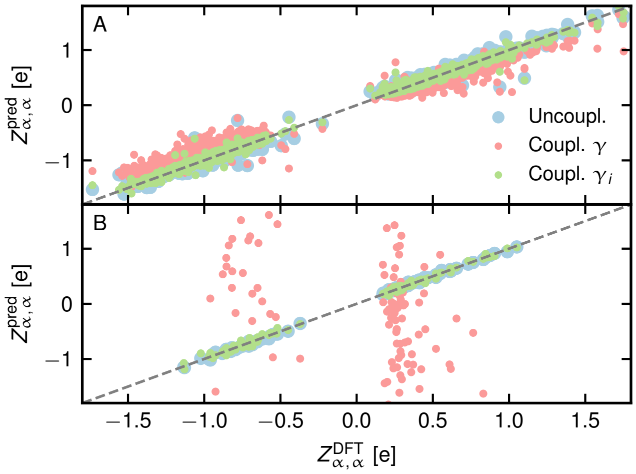


FIG. S2: Scatter plot of diagonal components of the BECs comparing DFT labels with model predictions. A shows bulk water and B water clusters.

## III. ANALYSIS OF PREDICTIONS FOR THE LOREM ARCHITECTURE

Figure S2 presents a scatter plot comparing diagonal components of the predicted BEC to DFT labels for bulk and cluster structures, analogous to Fig. 2 in the main text. The overall trends agree with those reported for the physical architecture: the uncoupled and local $\gamma_i$ model match the DFT labels well, whereas the global $\gamma$ model struggles to simultaneously fit bulk and cluster structures. For this global screening model, the bulk exhibits slightly smaller errors, whereas the clusters show larger deviations.

The fitted global $\gamma$ for bulk and cluster structures for a LOREM based global screening coupled model was determined to be $\gamma_{\mathrm{LOREM}} = -0.189$. This value, as well as fig. S3 highlight that the LOREM architecture's pseudo charges are no longer physically interpretable, but yield analogous results to the "physical models" shown in the main text.

Figure S4 shows—analogous to Fig. 5 in the main text—the imaginary part of the complex susceptibility spectrum for bulk water and two water hexamer cluster configurations, but now calculated with the LOREM architecture. The spectra show the same trends as the physical architectures, with the bulk spectrum matching experimental references well and the cluster spectra showing distinct features depending on the cluster geometry. Furthermore, we also find the global screening model to agree qualitatively with the other two models (and the experimental spectrum), but to struggle with reproducing the peak heights, which are reproduced more accurately with local screening and uncoupled models.

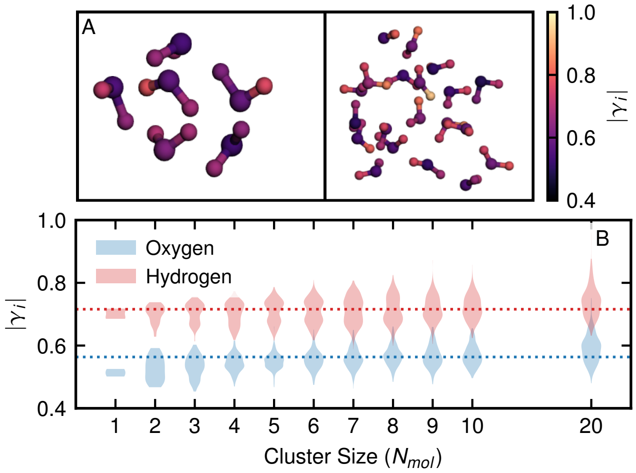


FIG. S3: A: Snapshot of water clusters with 6 and 20 atoms. Atoms are colored according to their local screening values $\gamma_i$. An interactive view is provided online `https://doi.org/10.24435/materialscloud:fs-8h`. B: Distribution of the local screening values from the physical coupled $\gamma_i$ model as a function of water cluster sizes. The average bulk values are given by dashed lines.

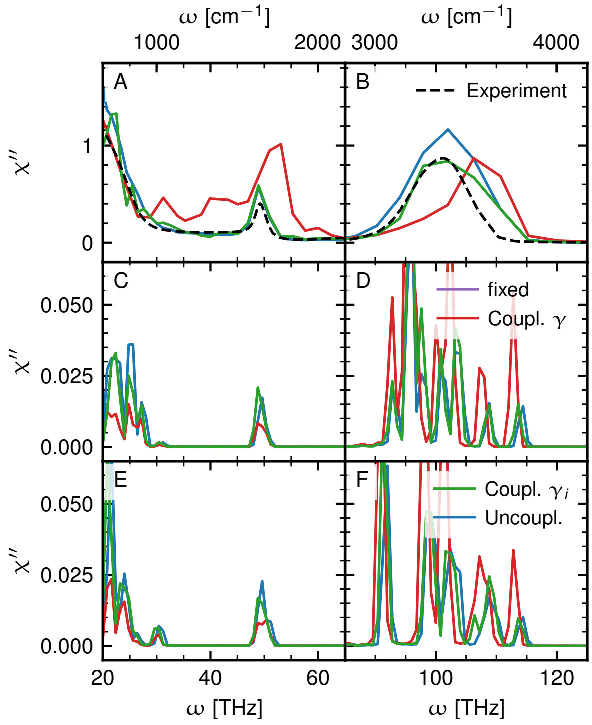


FIG. S4: Imaginary part of the complex susceptibility spectrum of periodic bulk water at $T = 300\,\mathrm{K}$ (A), a cage (B) and book (C) configuration of water hexamer cluster at $T = 10\,\mathrm{K}$. The black dashed line shows experimental bulk reference spectrum taken from Ref. 4.

## IV. STABILITY OF FINITE DIFFERENCES ESTIMATES FOR THE BECS

Figure S5 shows the stability of finite differences estimates for the BECs as a function of applied external fields $\boldsymbol{E}_{\mathrm{ext}}$ in a finite differences scheme. We find that over almost 9 orders of magnitude of applied field strengths, the estimates for the BECs remain very stable, with only a slight change for very small field strengths due to numerical noise.

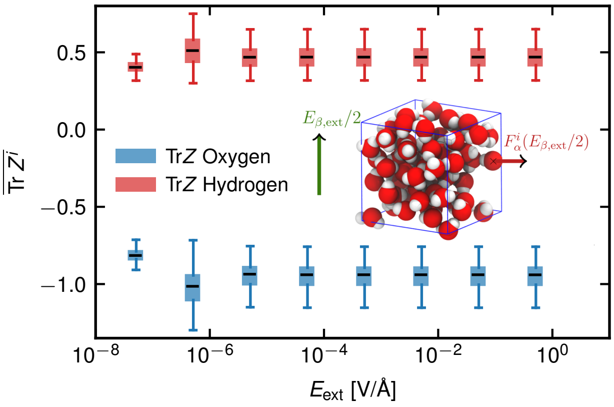


FIG. S5: Stability of finite differences estimates for the BECs as a function of applied fields $\boldsymbol{E}_{\mathrm{ext}}$ in a finite differences scheme. The boxplots show median, first to third quartile (box) and 1.5 times the inter-quartile range (whiskers) of BEC traces in order to give an estimate of the spread in values of the BECs. The schematic inset indicates how the finite field measurements are performed. One can clearly see that estimates are very stable across a wide range of field strengths.

---

[1] P. Zhong, D. Kim, D. S. King, and B. Cheng, *Machine learning interatomic potential can infer electrical response* (2025), 2504.05169.
[2] M. F. Langer, J. T. Frank, and F. Knoop, The Journal of Chemical Physics **159**, 174105 (2023), ISSN 0021-9606.
[3] M. F. Langer, Physical Review B **108** (2023).
[4] S. Carlson, F. N. Brünig, P. Loche, D. J. Bonthuis, and R. R. Netz, The Journal of Physical Chemistry A **124**, 5599 (2020), ISSN 1089-5639.
